# Synthesis of Micro-Spikes and Herringbones Structures by Femtosecond Laser Pulses on a Titanium Plate—A New Material for Water Organic Pollutants Degradation

**DOI:** 10.3390/ma14195556

**Published:** 2021-09-24

**Authors:** Joanna Kisała, Iaroslav Gnilitskyi, Bogumił Cieniek, Piotr Krzemiński, Michał Marchewka, Adriana Barylyak, Yaroslav Bobitski

**Affiliations:** 1 College of Natural Sciences, University of Rzeszow, Pigonia 1, 35-959 Rzeszow, Poland; bcieniek@ur.edu.pl; 2 Department of Photonics, Lviv Polytechnic National University, 1 Sviatoho Yura Sq., 79013 Lviv, Ukraine; iaroslav.gnilitskyi@novinano.com; 3 NoviNano Lab LLC, Paternaka Str. 5, 79000 Lviv, Ukraine; 4 Center for Microelectronics and Nanotechnology, Institute of Physics, University of Rzeszow, Pigonia 1, 35-959 Rzeszow, Poland; pkrzeminski@ur.edu.pl (P.K.); mmarchewka@ur.edu.pl (M.M.); 5 Department of Therapeutic Dentistry, Danylo Halitsky Lviv National Medicinal University, Pekarska Str. 69, 79010 Lviv, Ukraine; adriana.barylyak5@gmail.com

**Keywords:** femtosecond laser processing self-organized microstructures, herringbone structure, engineered materials, photocatalysis, waste water treatment

## Abstract

(1) Background: The shrinkage of water resources, as well as the deterioration of its quality as a result of industrial human activities, requires a comprehensive approach relative to its protection. Advanced oxidation processes show high potential for the degradation of organic pollutants in water and wastewater. TiO_2_ is the most popular photocatalyst because of its oxidizing ability, chemical stability and low cost. The major drawback of using it in powdered form is the difficulty of separation from the reaction mixture. The solution to this problem may be immobilization on a support (glass beads, molecular sieves, etc.). In order to avoid these difficulties, the authors propose to prepare a catalyst as a titanium plate covered with an oxide layer obtained with laser treatment. (2) Methods: In the present work, we generated titanium oxide structures using a cheap and fast method based on femtosecond laser pulses. The structurized plates were tested in the reaction of methylene blue (MB) degradation under UVA irradiation (365 nm). The photocatalytic activity and kinetic properties for the degradation of MB are provided. (3) Results: Studies of X-ray diffraction (XRD) and scanning electron microscopy (SEM) confirm a titanium oxide layer with laser-induced generated structures that are called “spikes” and “herringbones”. The structurized plates were effective photocatalysts, and their activity depends on the structure of the oxide layer (spike and herringbone). (4) Conclusions: The immobilization of the catalyst on a solid support can be performed in a fast and reproducible manner by using the technique of laser ablation. The layers obtained with this method have been shown to have catalytic properties.

## 1. Introduction

In recent years, there has been great concern over many serious environmental problems. The most important is the safety of water. There is a wide range of chemical and biological contaminants that may be found in water. The need for clean water places increased stress on the removal of organic pollutants and toxic heavy metal ions from water sources. There are several processes performed in order to improve water quality, such as chemical, physical, physico-chemical and biological processes. Chemical treatment includes precipitation, chemical disinfection, oxidation, advanced oxidation processes, and ion exchange [1].

Advanced oxidation technologies are based on in situ generation of strong oxidants, i.e., hydroxyl radicals, for the oxidation of organic pollutants. AOPs use strong oxidizing agents, such as hydrogen peroxide (H_2_O_2_) or ozone (O_3_), catalysts (iron ions, electrodes and metal oxides) and irradiation (UV light, visible-light and ultrasounds), separately or in combination under mild conditions (low temperature and pressure) [2]. Among different available AOPs, those driven by light seem to be attractive because of the abundance of solar light in regions where water scarcity is high and due to their relatively low costs and high efficiencies.

Titanium (IV) oxide (TiO_2_) is commonly known as a photocatalyst for the photodegradation of organic pollutants [3]. By using heterogeneous catalysts in wastewater treatment, two key problems are encountered: (i) the efficiency of the catalytic reaction and (ii) recovering photocatalyst after treatment. TiO_2_ is usually applied in suspension mode, resulting in a high surface-area-to-volume ratio. However, recovering this TiO_2_ powder slurry from treated wastewater requires filtration. Isolation of TiO_2_ slurry may clog the filter membrane, and catalyst particles will eventually pass through the porous filter, rendering the recovery process less efficient. The solution to this problem may be the immobilization of powder on a support (glass beads, molecular sieves, etc.). Hence, the use of a bulk-supporting substrate with a directly grown oxide layer is a reasonable choice to eliminate powder drawbacks.

The appearance of non-thermal ablation mechanisms much advanced surface modifications by femtosecond lasers [4]. It rendered these lasers attractive for surface treatments, which are aimed at the absence of heat-induced material damage on semiconductors, dielectric and metallic materials [5]. Apart from laser ablation, linearly polarized femtosecond laser pulses enable the formation of self-organised nanostructures, which are so-called laser-induced periodic surface structures, i.e., LIPSS [6]. LIPSS, otherwise known as surface ripples or nano gratings, are parallel periodic grooves produced on the surface of materials by laser irradiation, having a period proportional to the laser’s wavelength. Such sub-microstructures and nanostructures have a huge potential of application in such fields as tribology [7,8] and wettability [9,10,11,12,13] given their ability to improve the adhesion strength [13], as well as by improving differentiation of tissue cells [13,14,15], and surface colorization [10].

Aside from LIPSS, the femtosecond lasers allow the formation of the various types of nano/microstructures by tuning their parameters. For example, hexagons [16], grooves [6] and spikes [17] were formed by using of various number of laser pulses and tuning laser fluence. By changing the angle of incidence for laser light or a laser wavelength, the period of structures can be determined. Moreover, by varying laser fluence, the height and morphology can be changed, whereas the structure direction may be tuned by optics configuration. To summarize, the femtosecond nano- or microstructuring is a one-step process, which does not require vacuum or other arrangements and which proofs the flexibility and applicability relative to original materials for various purposes. During femtosecond laser processing and formation on the surface, self-organized structures form an oxidative layer. The formation of TiO_2_ and ZrO while LIPSS creation occurrence was observed in [18]. 

Tsukamoto et al. [19] reported the formation of cone-like microstructures on a titanium (Ti) plate by femtosecond laser ablation. Wavelength, pulse length and repetition rates were 800 nm, 100 fs and 1 kHz, respectively. The number of laser pulses varied from 10 to 230. The experiment was performed under vacuum. The period of the periodic nanostructures was about 700 nm. The spike structures were also obtained by Heitz et al. [20] in their experiments, where Ti-alloy substrates were irradiated in the air by Ti:sapphire femtosecond laser pulses (790 nm, 30 fs and 1 kHz). The spike structures were covered by more regular parallel submicrometric LIPSS grating structures, resulting in a hierarchical surface morphology. The periodicity of the LIPSS grating is about 300–350 nm. Liang et al. [21] fabricated a hierarchical TiO_2−x_ photoelectrode directly in situ on a metal substrate through femtosecond laser processing and anodization. Laser processing experiments were conducted using Ti:sapphire laser pulses (800 nm, 1 kHz) in an atmospheric environment. The obtained structure comprises spikes covered with large quantities of nanotubes. Huang et al. [22] fabricated nanoporous anatase TiO_2_ on a micro-structured Ti base by using a method involving three steps: femtosecond laser ablation, H_2_O_2_ oxidation and annealing. In their process, the Ti squares were scanned with a femtosecond laser (1030 nm, 800 fs and 400 kHz) in order to create different microstructures (triangular, square and conical).

All this suggests the simultaneous formation of metal oxides during femtosecond laser processing that substantially benefits photocatalytic properties.

There are a few reports dealing with the photocatalytic properties of laser structurized Ti surfaces. Liang et al. have already showed a method for fabricating hierarchical TiO_2−x_ photo electrodes by coupling femtosecond laser processing and anodization [21]. Anodization step caused the covering spikes by TiO_2_ nanotubes. This process narrowed the band gap (1.95 eV) and allowed visible light absorption. This hierarchical TiO_2−x_ photoelectrode demonstrated a higher than usual photodegradation rate of MB under visible light (k_app_ = 53 × 10^−3^ min^−1^, which was six times higher than those for P25—9 × 10^−3^ min^−1^). Extended light utilization (up to 636 nm) and synergetic action of spikes and nanotubes may cause excellent results (nanotubes served as a highway during the carriers’ transfer). In addition, the MB aqueous solution had a pH = 13.5, so the MB+ species are the only ones in the solution. The TiO_2_ surface is negatively charged, which increases the adsorption of dye on the catalyst surface. Other studies showing a photocatalytic property of spike-structurized Ti have been provided by Huang et al. [22]. They studied degradation of methyl orange. The structured Ti fabricated by femtosecond laser had superior light harvesting properties in the 200–1000 nm wavelength range, which was inherited by annealing TiO_2_, resulting in improved photocatalytic degradation of methyl orange under UV-light irradiation compared to nanoporous TiO_2_ fabricated by H_2_O_2_ oxidation and annealing only (without laser fabrication). In this paper, two types of self-organized microstructures were generated using high-power femtosecond laser pulses. The first type of microstructures consists of so-called “herringbone” structures, while the second type consists of typical “spike” structures. The photocatalytic properties of the obtained structures were tested in the methylene blue (model organic pollutant) degradation reaction. The novelty of the presented research is the lack of additional oxidation treatment (no chemical oxidation and annealing) and the use of the as-prepared materials for photocatalysis. Moreover, in the literature cited above, there is no mention of obtaining a herringbone structure on a titanium substrate.

## 2. Materials and Methods

### 2.1. Materials

In the present work, a commercially available titanium plate (0.6 mm thick) was cut into smaller pieces of 10 mm × 10 mm and cleaned with ethanol before laser treatment. Subsequently, these smaller plates were mechanically polished to mirror quality surfaces. All reagents were of analytical grade (Merck, Darmstadt, Germany) and were used as received.

### 2.2. Laser Surface Treatment

**Figure 1:**
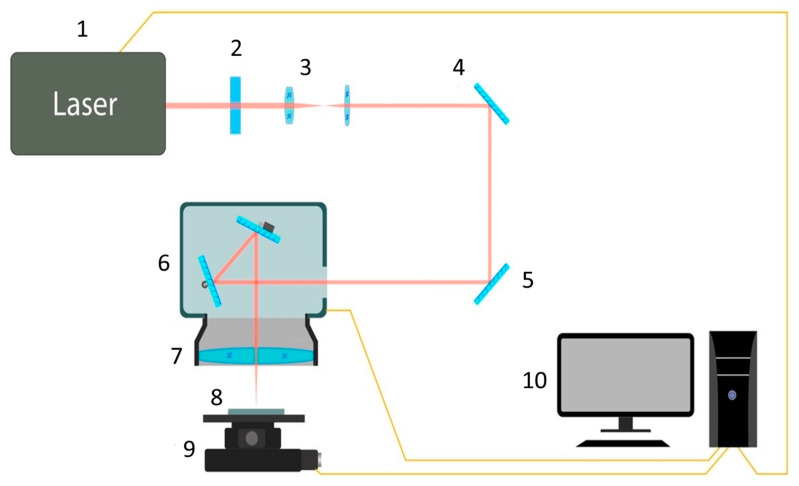
The scheme of the laser setup: laser (1), half-wave plate (2), beam expander (3), mirrors (4, 5), galvoscanner (6), F-Theta lens (7), sample (8) and six-axis positioner Standa (9), PC (10).

The generation of herringbone and spikes structures were conducted by using a femtosecond laser system “PHAROS” at its base wavelength of 1030 nm and with a pulse duration of 266 femtoseconds (Figure 1). The laser exposure was performed in the air. The power and uniformity of the laser beam were monitored by a “Standa” power meter and a high-speed detector, respectively. The spot size was estimated to be 22.67 (1/e^2^ of peak intensity).

The laser beam trajectory was controlled by the galvanic scanning head according to the preset in the micro-controller. An F-theta lens was used, which allowed processing large area samples without deviating from the focus. The square-shaped samples were processed by a focused laser beam on a 6-axis XYZ motorized table. All laser parameters were summarized in Table 1.

### 2.3. Photocatalytic Experiments

Photocatalytic properties evaluation was performed using methylene blue (MB) as the model compound. The Ti plates (P_a or P_b) were placed in a quartz cuvette, where MB solution was added (5 × 10^−5^ mol dm^−3^, pH = 6). The MB solution without catalyst plate was irradiated parallelly to measuring MB photolysis.

Twelve 9 W lamps (the number of photons per second in a beam ca. 1.84 × 10^18^) held at 30 cm from the sample with a 365 nm wavelength were used as the light sources. The catalytic experiment was carried out in six reaction systems summarized in Table 2. The methylene blue decay was monitored spectrophotometrically (UV-Vis 3100 PC spectrophotometer, VWR Ltd., Lutterworth, UK).

## 3. Results and Discussion

Scanning electron microscope (SEM, Vega3, Tescan, Brno, Czech Republic) operating at 30 kV and equipped with energy-dispersive X-ray detector (EDS, Oxford Instruments, High Wycombe, UK) operating at 15 kV was used to obtain the images of morphology and elemental composition of prepared surfaces. The analysis was carried out over the entire sample area. The elemental composition of samples was assessed by semi-quantitative analysis (resolution of analysis 1%). The chemical composition of the outer layer consists mainly of oxygen and titanium (Table 3).

**Figure 2:**
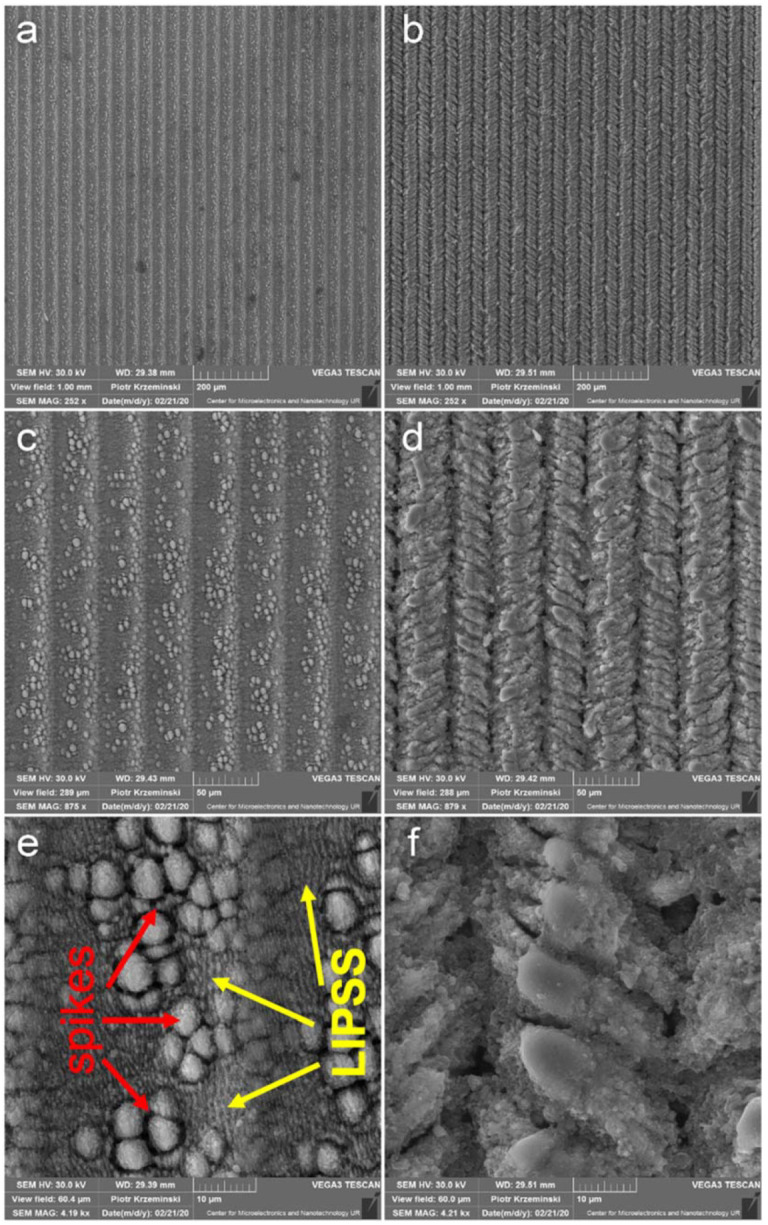
SEM images of surface structurized plates (ETD detector with SE mode); “spikes” structures—P_a (**a**,**c**,**e**); and herringbone structures—P_b (**b**,**d**,**f**).

The crystal phases were analyzed using ax X-ray diffractometer (XRD; D8 Advance, Bruker, Ettlingen, Germany) with Cu Kα (λ = 0.154056 nm) radiation, operated at 40 kV and 36 mA. Scanning electron microscopy (SEM) images of self-organized nanostructures and microstructures are shown in Figure 2a–f at different magnifications. The titanium plates were treated at 1030 nm wavelength with a focused beam (10 μm at 1/e^2^) scanning the sample surface with two different laser presets, which gave rise to structures named P_a (Figure 2a,c,e) and P_b (Figure 2b,d,f). Laser treatment results in the formation of LIPSS structures on the periphery of the laser spot, while so-called self-organized “spike” structures appear in the central part since the laser beam has a Gaussian shape (Figure 2a,c,e). “Spikes” are self-organized structures that have spherical form in the micrometer scale generated upon polarized ultrashort pulses with energy per pulse well above the ablation threshold. Another condition to form spikes is high repetition rate to maintain the heat accumulation process. Such heat accumulation results in complex hydrodynamic processes, which is also suggested by Tsibidis et al. [23]. The period of LIPSS is around 800 nm while the size of a spike is equal to 7–8 microns. Thus, P_a structures can be characterized as heterostructure between nanostructures and microstructures. P_b laser treatment results in the formation of “herringbone” structures (Figure 2b,d,f), which were firstly demonstrated in the work of Garcell et al. [24]. The herringbone structures constitute a singular channel with angled and axially symmetric ripples. Their period is around 10 microns. Comparison of our results and Huang’s [22] results suggests that increasing the repetition rate (>400 kHz) may have resulted in the formation of herringbone structures. The oxide layers were formed on the surface of Ti plates during the laser ablation in the air, and no further oxidative treatment was performed.

**Figure 3:**
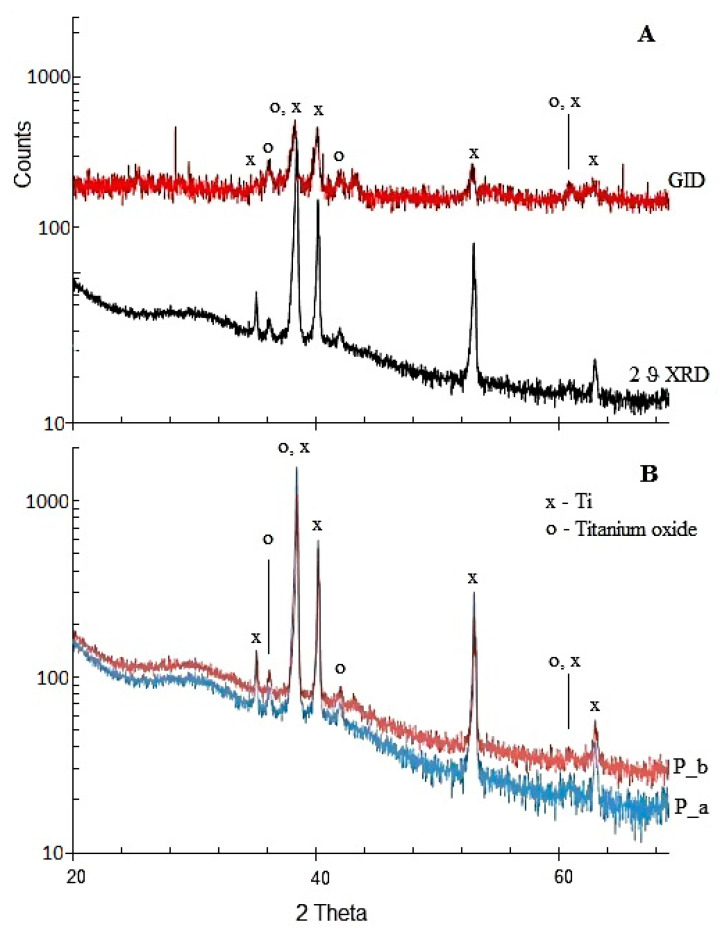
XRD measurements of the following: P_a—sample with “spikes”; P_b—sample with “herringbones”. Comparison of GID and 2θ XRD of P_b sample (**A**). Crystalline phases analyses in samples: P_a—spikes; P_b—herringbones (**B**).

The presence of crystalline reflexes in XRD patterns of P_a and P_b samples indicates their crystalline nature (Figure 3B). The observed XRD reflexes can be assigned to Ti (reflexes at 38, 40 and 52.5 °2θ) and TiOx (TiO and TiO_2_) (36, 38, 41, 42, 53, 61 and 63 °2θ) (Figure 3B). No reflections from other phases were observed. The reflexes’ assignation was carried out with the ICDD PDF database [25] and crystallographic open database COD [26]. The Grazing Incidence Diffraction (GID-XRD) allows the study of the surface layer covering the Ti plate. The observed reflexes (36, 42 and 61 °2θ) indicate mainly cubic TiO (COD 1536851 and F3m225) in the surface layer (Figure 3A). Different levels of P_a and P_b crystallinity were observed: ~70% (P_a) and ~80% P_b, respectively (measured in the angle range: 20–69 °2θ).

EDS measurements showed that the surface of P_b has a higher amount of oxygen atoms than the P_a surface. The SEM images (Figure 1) show differences in the probe’s surface morphology. P_b shows deeper penetration of the titanium plate than the P_a probe; hence, a different composition of the surface layers may be explained.

Catalytic experiments were carried out in six reaction systems in order to investigate the effect of the presence of the plate’s surface structure on the rate of MB degradation reaction. The photodegradation rate constant (k_app_) was determined for each degradation system with the assumption that the ongoing reactions were of the first order from Equation (1) [27]:

ln (C/C_0_) = −k_app_ t (1)

where k_app_ is the apparent rate constant; C_0_ and C are the initial concentration and concentration at time t.

**Figure 4:**
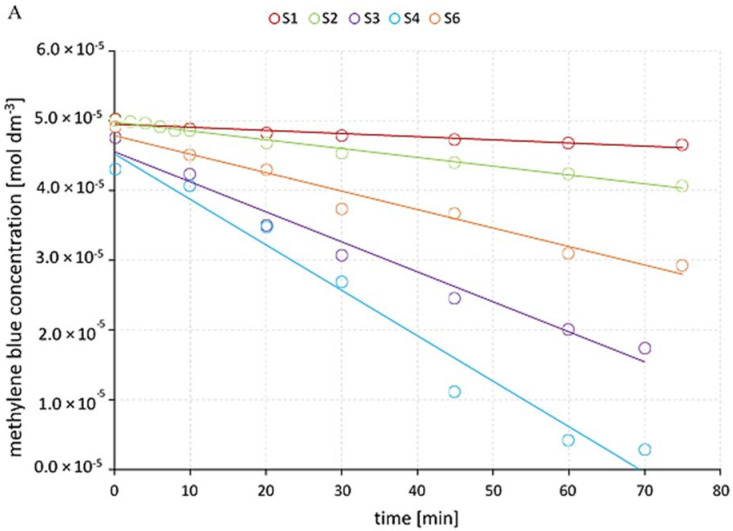

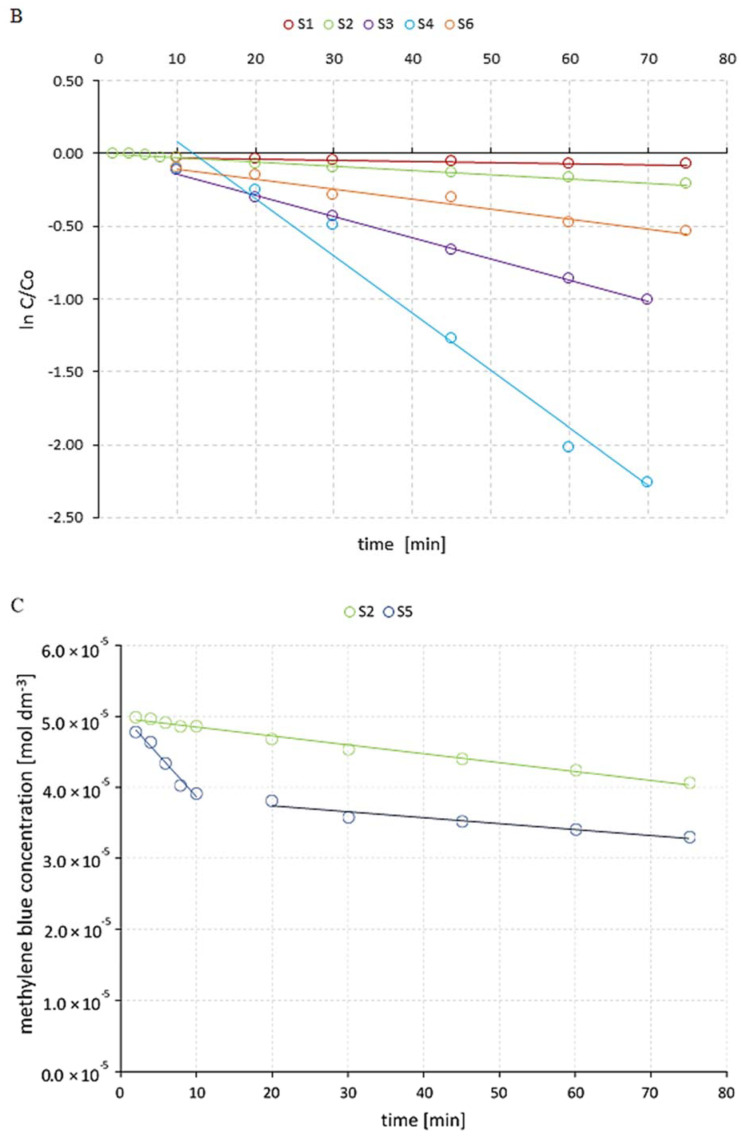

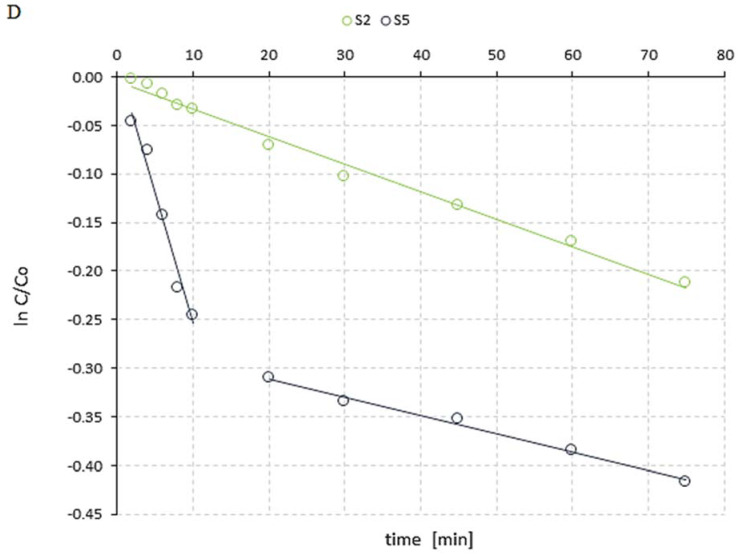
The methylene blue decay rate in all reaction systems (**A**); degradation kinetics of MB in studied reaction systems (**B**); MB decay rate on plates (**C**); MB degradation kinetics on plates (**D**).

A plot of ln C_0_/C versus time represents a straight line, as shown in Figure 4B, where the slope upon linear regression equals the apparent first-order rate constant k_app_. The obtained degradation rates are summarized in Table 4

**Figure 5:**
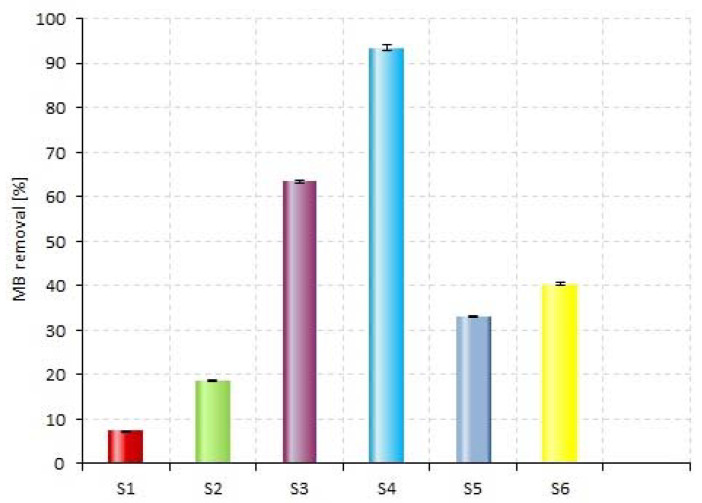
MB removal efficiency in all reaction systems; see Table 2 for description.

MB was photolyzed by direct UV radiation only up to 7.4% in 75 min in the absence of a catalyst (S1, Figure 5). MB irradiation in the presence of P_b caused an increase in MB removal up to 19% (S2 and Figure 5). Comparison of the apparent reaction rate constants (k_app_) of S1 and S2 show that the degradation of MB in the catalyst’s presence (S2) is 4.7 times faster than the photolysis reaction (S1) (Table 3 and Figure 5). Additional H_2_O_2_ increases the MB degradation efficiency in both systems—without P_b up to 63.5% (S3) and with plate up to 93.5% (S4). The reaction rate of S4 is almost three times that of S3, and it was 47 times that of S1. The degradation efficiency in the presence of P_a is lower in both S5 (33%) and S6 (40.5%) (Figure 5). Miscellaneous MB decay profiles observed for S2 and S5 systems suggest a different reaction route on the surface of the catalysts P_a and P_b.

**Figure 6:**
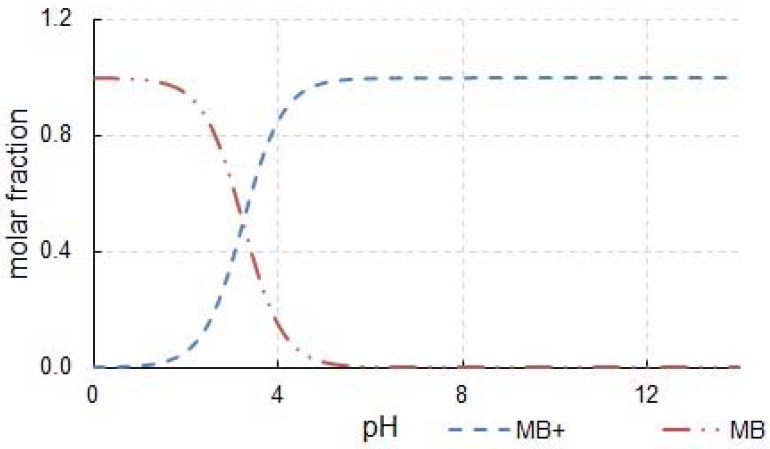
Distribution of MB ionic species depending on the solution pH.

The pH of the reaction system may strongly affect the degradation efficiency. The MB can be present in an aqueous solution as the cationic species (MB+) and undissociated molecules (MB). The speciation diagram of the MB is shown in Figure 6. As observed in this figure, the MB species predominates (80%) at pH = 2.7, both MB (50%) and MB+ (50%) species coexist at pH = pKa = 3.14 and MB+ is practically the only species present at pH ≥ 5.5.

For metal oxide surfaces, the H+ and OH^−^ are the charge determining ions [28]. Metal oxides develop a sizable positive surface charge when immersed in water of sufficiently low pH (Equation (2)). Titanium oxide surface at pH = 6 has a positive charge, which may decrease the adsorption of the dye.

TiOH + H^+^ → (TiOH_2_)^+^ (2)

The pH may also influence hydroxyl radical generation. There are two possible methods for the generation of hydroxyl radicals in photocatalysis: the first is generation from H_2_O_2_ by reaction with surface trapped electrons and the second is by reaction of OH^−^ with surface trapped holes [29]. The catalytic tests were performed with the addition of H_2_O_2_ as a source of hydroxyl radicals. At very acidic pH values, H_2_O_2_ is stabilized as H_3_O_2_^+^, which is deprotonated and subsequently disproportionates into O_2_ and H_2_O [30]. In order to avoid the problems mentioned above, pH = 6 was established for photocatalytic tests. This value was obtained after MB dissolved in distilled water (no additional substances were introduced into the reaction system).

A loss of MB at time zero indicates dye adsorption relative to the catalyst surface. In the case of the P_a plate, the loss is 1.96%, while it is 0.425% in the case of the P_b plate. The observed MB+ adsorption suggests that the P_a surface charge is more negative, while the P_b surface is more positive. Hence, the higher efficiency of MB decomposition on the P_a plate. In the case of the reaction S4 and S6, we observed a lower MB degradation efficiency on the P_a plate, which is probably due to the lower production of hydroxyl radicals on the catalyst surface. Adsorbed MB+ reduces the surface availability for H_2_O_2_. Hence, the smaller amount of •OH radicals produced.

The results of the EDS analysis showed that the stoichiometry of layer P_a corresponds to the sum formula TiO_0.75_, while the surface P_b has a different stoichiometry with higher oxygen content (probably consists of a mixture of oxides). The cubic compounds TiO_x_ have a broad homogeneity range with x varying from about 0.75 to 1.30 and the total vacancy content varying between 11 and 20%. The cation and anion vacancy concentrations change continuously over this range in an orderly fashion [31]. In oxygen-deficient TiOx films, oxygen vacancy energy states are formed below the conduction band minimum [32]. This results in a reduction in the energy required for photoexcitation of electrons and enhancement of the photocatalytic properties. Liang et al. [23] reported high efficiency of MB photodegradation in their work. The estimated k_app_ value was 53 × 10^−3^ min^−1^ (pH = 13.5), whereas the value obtained by us in system S4 was 43 × 10^−3^ min^−1^. This degradation system seems to be as efficient as the one presented by Liang.

The results of the catalytic tests carried out by us and presented in the literature cannot be compared due to the different composition of the surface layers and completely different conditions of the catalytic processes.

## 4. Conclusions

The surface of Ti plates was modified by using one-step process and ultrafast and relatively inexpensive methods based on using linearly polarized femtosecond laser pulses. By using the laser, the generated structures comprised titanium oxide layers as “spikes” (P_a) and “herringbones” (P_b). A thorough photocatalytic characterization was performed by using methylene blue (MB) as a model pollutant. The photocatalytic degradation of MB follows the pseudo-first-order reaction kinetics. The MB adsorption on structurized surfaces was estimated as losing MB at time zero (before irradiation starting) (MB adsorbed better on P_a than P_b). The results show that the best efficiency of MB degradation was on P_a (systems without H_2_O_2_, S2 and S5), whereas in systems with H_2_O_2_ (S4, S6) it was on P_b. This consequence indicates the MB degradation in S2 and S5 systems takes place on the P_a surface, while it takes place in the bulk of solution as a result of reaction with •OH radicals in the S4 and S6 systems. The efficiency of MB removal also confirmed this in the S3 system (MB photolysis in the presence of H_2_O_2_).

The immobilization of the catalyst on a solid support can be performed in a fast and reproducible manner by using the technique of laser ablation. The layers obtained in this manner have been shown to have catalytic properties. Using other post-structuring processes results in a different composition of the surface layer on the titanium plate.

## Figures and Tables

**Table 1:** Laser parameters used in an experiment.

Sample	Energy per Pulse, Ep (μJ)	Repetition Rate, RR (kHz)	Step (µm)	Scanning Speed, Vs (m/s)	Polarization	Pulse Duration, τ (fs)
P_a	0.9	200	10.5	1.05	⊥	266
P_b	5	1000	9	0.12	⊥	266

**Table 2:** Photocatalytic reaction systems.

Reaction Systems		H_2_O_2_ Concentration (%)
Signature	Description	
S1	MB + light	0
S2	MB + P_b + light	0
S3	MB + H_2_O_2_ + light	1.14
S4	MB + H_2_O_2_ + P_b + light	1.14
S5	MB + P_a + light	0
S6	MB + H_2_O_2_ + P_a + light	1.14

**Table 3:** The estimated elemental concentration (atomic percentage) in the outmost layer of Ti plates determined by EDS.

Sample	Ti	O
Ti plate (control)	84	16
P_a	80	20
P_b	46	54

**Table 4:** Kinetic parameters of catalytic reactions.

Reaction System	k_app_ (min^−1^)	t_1/2_ (min)
S1	0.9 × 10^−3^	770.1
S2	4.2 × 10^−3^	165.0
S3	14.6 × 10^−3^	47.8
S4	43 × 10^−3^	16.4
S5	27.6 × 10^−3^	25.11
S6	6.8 × 10^−3^	101.9

## Data Availability

Data available on request due to restrictions e.g., privacy or ethical. The data presented in this study are available on request from the corresponding author. The data are not publicly available. The data are not publicly available because they are part of the ongoing project.
